# Association between oral health and advisability of oral feeding in advanced cancer patients receiving palliative care: a cross-sectional study

**DOI:** 10.1007/s00520-022-06984-w

**Published:** 2022-03-28

**Authors:** Junichi Furuya, Hiroyuki Suzuki, Rena Hidaka, Chiaki Matsubara, Yuko Motomatsu, Yuji Kabasawa, Haruka Tohara, Yuji Sato, Satoshi Miyake, Shunsuke Minakuchi

**Affiliations:** 1 Department of Geriatric Dentistry, Showa University School of Dentistry, 2-1-1 Kitasenzoku, Ohta-ku, Tokyo, 145-8515 Japan; 2 Department of Dysphagia Rehabilitation, Graduate School of Medical and Dental Sciences, Tokyo Medical and Dental University (TMDU), 1-5-45 Yushima, Bunkyo-ku, Tokyo, 113-8549 Japan; 3 Department of Gerodontology and Oral Rehabilitation, Graduate School of Medical and Dental Sciences, Tokyo Medical and Dental University (TMDU), 1-5-45 Yushima, Bunkyo-ku, Tokyo, 113-8549 Japan; 4 Department of Oral Health Sciences for Community Welfare, Graduate School of Medical and Dental Sciences, Tokyo Medical and Dental University (TMDU), 1-5-45 Yushima, Bunkyo-ku, Tokyo, 113-8549 Japan; 5 Department of Nursing, Tokyo Medical and Dental University Hospital, 1-5-45 Yushima, Bunkyo-ku, Tokyo, 113-8549 Japan; 6 Department of Oral Care for Systemic Health Support, Graduate School of Medical and Dental Sciences, Tokyo Medical and Dental University (TMDU), 1-5-45 Yushima, Bunkyo-ku, Tokyo, 113-8549 Japan; 7 Center for Innovative Cancer Treatment, Tokyo Medical and Dental University Hospital, 1-5-45 Yushima, Bunkyo-ku, Tokyo, 113-8549 Japan

**Keywords:** Palliative care, Nutrition, Oral health, Swallowing, Dysphagia

## Abstract

**Purpose:**

Maintenance of oral feeding is important in terms of maintaining and improving the quality of life in terminal cancer patients receiving palliative care. Although adequate oral health status is essential for oral feeding in hospitalized patients, the relationship between oral health and oral feeding in patients receiving palliative care remains unclear. This cross-sectional study aimed to examine how the general condition and oral health status of these patients relate to decisions regarding their nutritional intake methods.

**Methods:**

This retrospective cross-sectional study included 103 terminal cancer patients (59 men and 44 women; mean age, 73.8 ± 10.9 years) who received palliative care between April 2017 and August 2019. The nutritional method was assessed using the Functional Oral Intake Scale (FOIS). We assessed two types of nutritional methods: (1) the method advised by the attending physician until the initial dental examination (FOIS-I) and (2) the recommended method based on consultation with a palliative care doctor and dentist after the initial oral examination (FOIS-R). Furthermore, the participants’ basic information and Dysphagia Severity Scale (DSS) and Oral Health Assessment Tool (OHAT) scores were assessed.

**Results:**

There was a divergence between FOIS-I and FOIS-R. FOIS-R was significantly higher than FOIS-I (*p* < 0.001). Multiple regression analysis revealed that the time until death, DSS score, and OHAT score had a significant impact on determining the food form for oral feeding.

**Conclusions:**

Appropriate oral health assessment is important in determining the food form and indication for oral feeding among patients receiving palliative care.

## Background

Terminal cancer patients are provided palliative care through a multidisciplinary approach to improve their quality of life (QOL) through pain control and by comprehensively addressing the various forms of distress they experience, thereby facilitating a peaceful end-of-life experience [1]. In this type of palliative care, patient-tailored nutritional support can effectively improve their QOL [2, 3]. The nutritional support for patients receiving palliative care relies on nutritional counseling, dietary modifications, and supplementary nutrition and hydration through dietary supplements or intravenous drips [3, 4]. Among the available approaches, nutritional counseling is effective in increasing oral feeding and managing symptoms that impede oral feeding. Avoiding specific dietary restrictions and allowing patients to eat foods they enjoy as much as possible is also recommended [5]. Thus, in palliative care, maximizing patients’ food enjoyment and minimizing food-related discomfort through nutritional support [2] aimed at maintaining oral feeding are extremely important [6] and are expected to help maintain or improve patients’ QOL. In general, a normal diet of solid food is preferred. Therefore, mastication and the status of the oral cavity contribute substantially to the ability to enjoy eating.

The main nutritional pathways for terminal cancer patients receiving palliative care are oral and enteral feeding [7, 8]. However, systemic symptoms [9], such as weakness, fatigue, lack of appetite, pain, and depression, make oral feeding difficult for many terminal cancer patients. Furthermore, because complications, such as gastrointestinal obstructions, can block the intestinal nutritional route, some patients require intravenous feeding [10]. Cachexia is a major nutritional problem in terminal cancer patients receiving palliative care [11]. It is reported that approximately 20% of patients in the terminal stage of cancer die due to cachexia rather than cancer itself [12]. Moreover, cachexia causes symptoms that affect oral intake, such as loss of appetite, taste changes, fatigue, and sarcopenia, and significantly impairs the patient’s QOL. Therefore, it is important to provide appropriate nutritional and oral interventions early on to prevent cachexia from developing into an irreversible condition. Oral feeding is useful to achieve effective nutritional intake, and it is important to maintain oral intake not only in terms of QOL but also in terms of nutrition in terminal cancer patients. One study [13] of inpatients at an acute care hospital demonstrated that in addition to consciousness level and activities of daily living (ADL), the factors affecting the chosen food form include tongue coating, which indicates tongue function and molar occlusal support status. These factors affect patients’ masticatory and swallowing ability. Additionally, at acute care hospitals, some patients are assigned a nutritional support team that includes dental professionals. In these patients, oral health status, including swallowing ability, and oral environmental factors, such as dentures and occlusion, influence the nutrition intake method [14, 15]. Based on these findings, good oral function and the intervention of dental professionals during palliative care are essential for the maintenance of oral feeding in terminal cancer patients. Moreover, these studies have also reported that a proper assessment of oral function can increase the number of hospitalized in-patients who can receive food orally [13–15].

Terminal cancer patients receiving palliative care are likely to experience oral-related problems before death. These problems often involve symptoms of oral discomfort associated with oral feeding, such as oral dryness, dysphagia, dysgeusia, and tongue-coating adhesion [16, 17]. The awareness of the importance of oral health care for patients receiving palliative care is growing because adequate oral health status is essential for oral feeding. Dental professionals are expected to participate and implement appropriate oral health care as a member of the multidisciplinary team providing palliative care. However, because little scientific evidence exists regarding the relationship between oral health status and oral feeding or the discrepancies between oral feeding capability and nutritional methods observed in inpatients receiving terminal care, the role of dental professionals in palliative care is unclear. If the relationship between oral health and oral feeding is clarified in terminal cancer patients receiving palliative care, it will not only lead to the implementation of appropriate interventions by dental professionals, but also to the realization of a higher level of nutritional intake, which will ultimately contribute to the maintenance and improvement of their nutritional status and QOL. Therefore, we conducted a cross-sectional study of terminal cancer patients receiving palliative care to clarify the relationship between the advisability of oral feeding and oral health status. Moreover, this study aimed to elucidate the factors determining the food form when oral feeding is possible.

## Methods

### Participants

**Fig. 1:**
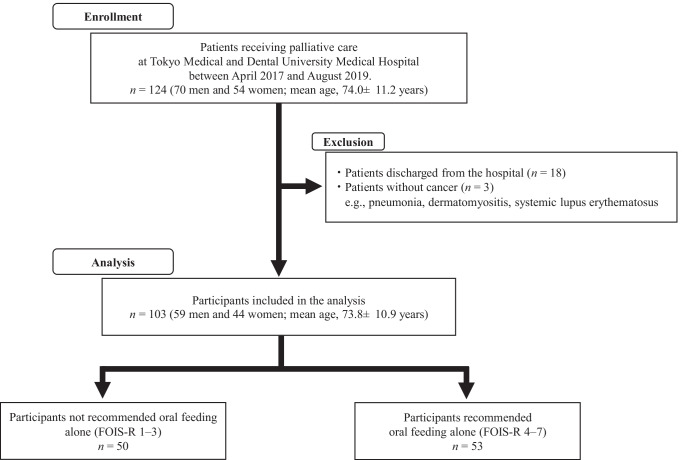
Enrollment process for this retrospective cross-sectional study. Abbreviation: FOIS-R Functional Oral Intake Scale after revision

Figure 1 shows the enrollment process of this retrospective cross-sectional study. This study enrolled 124 patients (70 men and 54 women; mean age, 74.0 ± 11.2 years) who received palliative care at Tokyo Medical and Dental University Medical Hospital (Tokyo, Japan) between April 2017 and August 2019 as potential participants. All outcomes were extracted retrospectively from the medical and dental records during palliative care. The inclusion criteria were as follows: patients with terminal cancer, patients with complaints of oral health problems voiced by themselves or by their family or nurses, and patients who died after receiving palliative care at our institute between April 2017 and August 2019. Considering these inclusion criteria, 21 patients were excluded. Finally, this study included 103 participants (59 men and 44 women; mean age, 73.8 ± 10.9 years) who received palliative care for terminal cancer. In this study, an opt-out method was used to obtain consent for participation. Prior to the start of this study, a research explanation document that clearly described the present study, including the use of data containing anonymized medical information was posted on our hospital website, so that the participants had the opportunity to refuse to participate in the study. This study was approved by the ethical review board of the Faculty of Dentistry at Tokyo Medical and Dental University (approval no. D2016-077).

### Outcomes

#### Nutritional method assessment

The nutritional method was evaluated using the Functional Oral Intake Scale (FOIS) [18]. A previous study revealed a discrepancy between the actual nutritional method used and the recommended nutritional method based on the patients’ general and oral condition [13]. Therefore, we conducted two 7-point assessments: the nutritional method followed by the attending medical doctor until the first oral examination (i.e., FOIS at the initial examination, [FOIS-I]) and a modified recommendation after the oral examination (i.e., FOIS after revision [FOIS-R]). The FOIS-R score was determined after a dentist conducted a comprehensive evaluation of the oral health status (including swallowing function) and after a consultation with a palliative care doctor to consider the participant’s overall condition.

#### Oral health assessment

The oral outcomes included the swallowing ability based on the Dysphagia Severity Scale (DSS) [19] and a comprehensive oral health evaluation using the Oral Health Assessment Tool (OHAT) [20]. The number of teeth referred to the number of remaining natural teeth, and the number of functional teeth included prosthetic teeth, such as dentures or implants. Residual roots were excluded from both counts. Oral outcomes were evaluated at the bedside by a dentist who was a fully trained member of the palliative care team.

The DSS is a tool that comprehensively evaluates the severity of dysphagia in seven stages: the lower the score, the more severe the dysphagia. OHAT is a tool for assessing a patient’s oral cavity by evaluating the lips, tongue, gums, mucous membranes, saliva, remaining teeth, dentures, oral cleaning, and tooth pain in three stages (i.e., 0–2). High OHAT scores indicate a poor oral health status.

#### Other independent variables

Basic data on age, sex, primary cancer site, the time until death from dental intervention, ADL, and consciousness level were retrospectively obtained from the medical records. The time until death was evaluated at four stages (number of days from the initial dental examination to death): within 1 week (0), 2–4 weeks (1), 1–2 months (2), and over 3 months (3). This classification was chosen based on previous studies, which reported that the terminal stage of cancer generally lasts 1–2 months [21] and that ADL rapidly declines 2–3 weeks before death [22]. ADL was evaluated at five levels, based on the performance status (PS) [23]: “0” = the ability to engage in activities without any problems and to live without restrictions, such as before the disease; “1” = having limitations on intense physical activities but being able to walk and perform light work and tasks while sitting; “2” = being able to walk and take care of oneself but being unable to work, spending < 50% of the day in bed, and only being able to take care of oneself in limited ways; “3” = being unable to move, spending ≥ 50% of the day in bed or in a chair, and being unable to care for oneself; and “4” = spending all the time in bed or in a chair. Consciousness level was based on the Japan Coma Scale (JCS) [24], which uses the following four stages: lucid (0), awake (I), arousable with stimulus (II), and non-arousable even with a stimulus (III).

### Statistical analysis

The difference between the FOIS-I and the FOIS-R was compared using the Wilcoxon signed-rank test. For basic participant data and oral health status, the participants were separated into two groups, based on whether oral feeding, as the main nutrition method, was possible (FOIS-R 4–7) or not (FOIS-R 1–3). Categorical variables were examined using the chi-square test. Ordinal and continuous variables were compared using the Mann–Whitney *U* test. To examine the factors affecting whether nutrition could be obtained by oral feeding alone, logistic regression analysis was conducted with the FOIS-R score as the objective variable (FOIS-R 1–3 = 0 and FOIS-R 4–7 = 1) and age, JCS score, the time until death, DSS, and OHAT total score as the explanatory variables. Furthermore, to examine the factors affecting the determination of food form in participants who could obtain nutrition by oral feeding alone (i.e., FOIS-R 4–7), multiple regression analysis was conducted using the FOIS-R score as the objective variable and age, JCS score, the time until death, DSS, and OHAT score as the explanatory variables. PS was excluded from the explanatory variables in the multivariate analysis because it was strongly associated with the JCS score. SPSS Ver. 27 (IBM Japan, Tokyo, Japan) was used for statistical analysis. The significance level was set at 5%.

## Results

### Discrepancies between the initial and recommended nutritional methods in terminal cancer patients

Table 1 shows the distribution of the initial and recommended nutritional methods (i.e., FOIS-I and FOIS-R, respectively) of the study participants. A significant difference was observed between FOIS-I (mean score, 3.6 ± 2.6; median score, 2) and FOIS-R scores (mean score, 3.8 ± 2.3; median score, 4; *p* = 0.024). Discrepancies between the FOIS-I and FOIS-R scores due to the lack of an appropriate comprehensive assessment of the oral health status were observed in 35 (34.0%) participants. Among participants with a discrepancy, 24 (68.6%) participants had a lower level of nutrition in relation to the recommended method, while for the remaining 11 participants (31.4%), a higher level than recommended was selected. Overall, 51.5% of the participants were recommended to obtain nutrition by oral feeding alone, after correcting for the nutritional method more suitable for the participant’s general condition and oral health.

**Table 1:** Nutritional method determined at the initial and revised evaluation

**FOIS**	**FOIS-I**		FOIS-R*	
	***n***	**%**	***n***	**%**
1	40	38.8	25	24.2
2	12	11.7	20	19.4
3	4	3.9	5	4.9
4	3	2.9	4	3.9
5	3	2.9	11	10.7
6	19	18.4	22	21.4
7	22	21.4	16	15.5

Distribution of nutrition methods from the initial dental exam (FOIS-I) and those recommended after assessing the general condition and oral health (FOIS-R). *p < 0.001, FOIS at intervention versus the recommended FOIS (based on the Wilcoxon signed-rank test)

*Abbreviations*: *FOIS* Functional Oral Intake Scale, *FOIS-I* FOIS at initial intake, *FOIS-R* FOIS after revision

### Factors affecting the ability to obtain nutrition by oral feeding alone

Table 2 compares the basic data and oral health status of participants based on whether the recommended nutritional method was solely oral feeding (i.e., FOIS-R 1–3 group [*n* = 50] vs. FOIS-R 4–7 group [*n* = 53]). Significant differences were observed between groups in the number of days between the initial oral examination and death (*p* = 0.001), DSS (*p* < 0.001), and OHAT total score (*p* < 0.001). With regard to the OHAT subscales, significant differences were observed between the groups for lips (*p* = 0.001), saliva (*p* < 0.001), and oral cleaning (*p* < 0.001). These results indicated that the group that could obtain nutrition from oral feeding alone (FOIS-R 4–7) had a better oral health status than those who had difficulty obtaining nutrition by oral feeding (FOIS-R 1–3).

**Table 2:** Characteristics of patients who could and could not obtain sufficient nutrition by oral feeding alone

		FOIS-R 1–3 Group				FOIS-R 4–7 Group				
		Mean ± SD	Median	*n*	%	Mean ± SD	Median	*n*	%	*p* value
Age		72.8 ± 11.0	74.5	50		74.8 ± 10.8	76	53		0.263
Sex	Male	-	-	31	62.0	-	-	28	52.8	0.347
	Female	-	-	19	38.0	-	-	25	47.2	-
Primary cancer site	Lung cancer	-	-	8	16.0	-	-	14	26.4	-
	Head and neck cancer	-	-	10	20.0	-	-	6	11.3	-
	Bladder cancer	-	-	6	12.0	-	-	1	1.9	-
	Colorectal cancer, Small intestine cancer	-	-	4	8.0	-	-	3	5.7	-
	Pancreatic cancer	-	-	3	6.0	-	-	4	7.5	-
	Kidney cancer	-	-	3	6.0	-	-	4	7.5	-
	Liver cancer	-	-	2	4.0	-	-	3	5.7	-
	Prostate cancer	-	-	2	4.0	-	-	4	7.5	-
	Gastric cancer	-	-	3	6.0	-	-	2	3.8	-
	Esophageal cancer	-	-	3	6.0	-	-	0	0.0	-
	Malignant lymphoma	-	-	1	2.0	-	-	2	3.8	-
	Other			5	10.0	-		10	18.9	-
Days from the first dental examination to death		17.4 ± 16.1	13	50		34.2 ± 29.6	22	53		0.001*
Time until death from dental intervention	Within 1 week	-	-	17	34.0	-	-	5	9.4	-
	2–4 weeks	-	-	22	44.0	-	-	24	45.3	-
	1–2 months	-	-	9	18.0	-	-	15	28.3	-
	Over 3 months	-	-	2	4.0	-	-	9	17.0	-
JCS score	0	-	-	10	20.0	-	-	34	64.1	-
	I	-	-	27	54.0	-	-	17	32.1	-
	II	-	-	12	24.0	-	-	2	3.8	-
	III	-	-	1	2.0	-	-	0	0.0	-
PS	0	-	-	0	0.0	-	-	0	0.0	-
	1	-	-	0	0.0	-	-	5	9.4	-
	2	-	-	1	2.0	-	-	14	26.4	-
	3	-	-	8	16.0	-	-	25	47.2	-
	4	-	-	41	82.0	-	-	9	17.0	-
DSS		2.9 ± 1.7	2	50		5.4 ± 1.0	6	53		< 0.001*
	1	-	-	11	22.0	-	-	0	0.0	-
	2	-	-	18	36.0	-	-	0	0.0	-
	3	-	-	3	6.0	-	-	1	1.9	-
	4	-	-	9	18.0	-	-	10	18.9	-
	5	-	-	2	4.0	-	-	15	28.3	-
	6	-	-	7	14.0	-	-	22	41.5	-
	7	-	-	0	0.0	-	-	5	9.4	-
OHAT total		6.9 ± 2.6	6.5	50	-	4.6 ± 2.4	4	53	-	< 0.001*
OHAT	Lips	0.6 ± 0.5	1	-	-	0.3 ± 0.6	0	-	-	0.001*
	Tongue	1.2 ± 0.6	1	-	-	1.0 ± 0.8	1	-	-	0.246
	Gums/Mucosa	0.8 ± 0.6	1	-	-	0.6 ± 0.7	0	-	-	0.058
	Saliva	1.5 ± 0.6	1.5	-	-	0.8 ± 0.6	1	-	-	< 0.001*
	Teeth	0.3 ± 0.6	0	-	-	0.3 ± 0.6	0	-	-	0.865
	Dentures	0.7 ± 0.9	0	-	-	0.5 ± 0.8	0	-	-	0.376
	Oral hygiene	1.3 ± 0.8	2	-	-	0.7 ± 0.7	1	-	-	< 0.001*
	Dental pain	0.5 ± 0.7	0	-	-	0.3 ± 0.6	0	-	-	0.280

Comparison of basic patient data and oral health status of participants who had difficulty obtaining sufficient nutrition by oral feeding alone (FOIS-R 1–3 group) and those who could obtain sufficient nutrition by oral feeding alone (FOIS-R 4–7 group). *p < 0.05; FOIS-R 1–3 versus FOIS-R 4–7, based on the Mann–Whitney *U* test

*Abbreviations*: *FOIS* Functional Oral Intake Scale, *FOIS-R* FOIS after revision, *SD* standard deviation, *JCS* Japan Coma Scale, *PS* performance status, *DSS* Dysphagia Severity Scale, *OHAT* Oral Health Assessment Tool

Table 3 shows the results of the logistic regression analysis with nutrition obtained from oral feeding alone as the objective variable (“0” = FOIS-R 1–3; “1” = FOIS-R 4–7). Obtaining nutrition from oral feeding alone was significantly associated with age (odds ratio [OR] 1.067; 95% confidence interval [CI] 1.004–1.134), time until death (OR 2.469; 95% CI 1.078–5.654), and DSS (OR 3.065; 95% CI 1.810–5.187).

**Table 3:** Logistic regression analysis to determine factors associated with obtaining sufficient nutrition by oral feeding alone

Independent variables	Odds ratio	95% confidence interval	*p* value
Age	1.067	1.004 to 1.134	0.036*
JCS	0.559	0.203 to 1.540	0.261
The time until death	2.469	1.078 to 5.654	0.033*
DSS	3.065	1.810 to 5.187	< 0.001*
OHAT	1.070	0.824 to 1.389	0.614

Number of participants evaluated in this model = 103. FOIS-R 1–3 = 0; FOIS-R 4–7 = 1; sex: male = 0, female = 1; time until death from the dental examination: within 1 week = 1, 2–4 weeks = 2, 1–2 months = 3, over 3 months = 4. The other independent variables were used as continuous variables. *p < 0.05, based on logistic regression analysis

*Abbreviations*: *FOIS* Functional Oral Intake Scale, *FOIS-R* FOIS after revision, *JCS* Japan Coma Scale, *DSS* Dysphagia Severity Scale, *OHAT* Oral Health Assessment Tool

### Factors impacting the determination of food form when nutrition can be obtained by oral feeding alone

Table 4 shows the results of multiple regression analysis using the FOIS score as the objective variable in participants who could obtain nutrition by oral feeding alone (FOIS-R 4–7). Time until death, DSS, and OHAT had a significant impact on determining the food form in participants who could obtain nutrition from oral feeding alone.

**Table 4:** Multiple regression analysis of the FOIS-R 4–7 group using FOIS-R as an objective variable

Independent variable	*B*	SE	95% confidence interval	*β*	*p* value	Variance inflation factor
Age	− 0.001	0.009	− 0.019 to 0.018	− 0.010	0.927	1.031
JCS	0.020	0.184	− 0.351 to 0.391	0.012	0.914	1.134
The time until death	− 0.279	0.123	− 0.527 to − 0.030	− 0.273	0.029*	1.257
DSS	0.442	0.118	0.205 to 0.679	0.470	< 0.001*	1.343
OHAT	− 0.129	0.051	− 0.231 to − 0.027	− 0.347	0.015*	1.606

Number of participants evaluated in this model = 53. Multiple *R* = 0.672; *R*^2^ = 0.451; *p* < 0.001. B, partial regression coefficient; SE, standard error; *β*, standardized partial regression coefficient; sex: male = 0, female = 1; Time until death from dental exam: within 1 week = 1, 2–4 weeks = 2, 1–2 months = 3, over 3 months = 4. All other independent variables were used as continuous variables.*p < 0.05, based on multiple regression analysis

*Abbreviations*: *FOIS* Functional Oral Intake Scale, *FOIS-R* FOIS after revision, *JCS* Japan Coma Scale, *DSS* Dysphagia Severity Scale, *OHAT* Oral Health Assessment Tool

## Discussion

In this cross-sectional study of terminal cancer patients receiving palliative care, we aimed to clarify the relationship between oral feeding status and oral health status and the factors associated with determining the food form when oral feeding is possible. The present results showed discrepancies between the nutritional method provided before an oral health evaluation and that recommended based on the patient’s general condition and oral health after evaluation. Moreover, the results suggested that when deciding whether obtaining nutrition by oral feeding alone is possible, good swallowing ability is important, independent of long prognosis. Furthermore, when oral feeding is possible, a healthy oral health status, in addition to a long prognosis and good swallowing ability, is necessary to select a higher level of food form. The clinical relevance of this study was to demonstrate that oral assessment by dental professionals as part of a multidisciplinary approach may lead to better selection of the appropriate nutritional intake methods for terminal cancer patients who show a variety of oral problems [16, 17].

Managing cachexia is an important part of the nutritional management of cancer patients. Cachexia is a complex syndrome of metabolic abnormalities with the primary symptom of loss of skeletal muscle mass against a background of inflammation due to chronic disease [11]. This condition has been observed in many terminal cancer patients. Cachexia may be caused by insufficient nutritional intake due to poor appetite or difficulty in oral feeding due to cancer or adverse events associated with treatment [25]. Common nonsurgical treatments for cancer patients are radiation therapy and chemotherapy and among their adverse effects are symptoms of oral discomfort, such as mucosal inflammation, stomatitis, dysphagia, and dysgeusia [26, 27], which can make oral feeding difficult. Enteral or intravenous feeding is often selected to ensure that these patients receive adequate nutrition [10]. In this study, enteral or intravenous feeding was selected as the main nutritional route for > 50% participants at the initial dental examination. Many participants did not receive oral feeding. Contrarily, when the participants’ general condition and oral health were appropriately assessed, the proportion of participants deemed capable of oral feeding increased, and approximately half were able to receive sufficient nutrition through oral intake alone. These results highlight the importance of the appropriate assessment of nutritional intake, including oral health assessment by dental professionals, in patients at the end stage of cancer.

Nutrition-related issues, such as difficulty with oral feeding and loss of appetite, are of great concern to terminal cancer patients. Minimizing unpleasant symptoms associated with food and maximizing food enjoyment through oral feeding is important in the palliative care of terminal cancer patients [6]. Dental services are an essential part of multidisciplinary approaches to palliative care [28]. In fact, it has been reported that palliative care patients with impaired oral health require specialized oral care by dental hygienists, dental caries treatment, and prosthetic treatment, including denture repair by dentists [17]. Therefore, the results of this study suggested that including appropriate oral health evaluations and interventions by dental professionals as a part of palliative care could be effective in eliminating unnecessary restrictions on oral feeding and maintaining or improving QOL for terminal cancer patients.

Ohno et al. [29] examined changes in oral feeding status in the 2 weeks before death in terminal cancer patients. They found that most patients became incapable of oral feeding 6 days before death. Additionally, when patients who could not eat orally due to intestinal tract problems were excluded, most of the remaining patients became incapable of oral feeding 4 days before death. Complaints concerning hunger and dry mouth disappear as death approaches [30, 31], and forcing these patients to continue oral feeding may increase stress levels. Thus, if a patient expresses a desire to eat, palliative care interventions should be provided to allow the patient to continue oral feeding as much as possible.

When deciding whether oral feeding is possible, a suitable evaluation of its safety is essential. A large proportion of terminal cancer patients receiving palliative care have dysphagia [32]. If oral feeding is forced on these patients, conditions, such as aspiration pneumonia and suffocation, could occur and greatly harm their QOL [33]. These findings indicate the importance of performing oral feeding safely and in consideration of their swallowing ability. Furthermore, when oral feeding is possible, in addition to issues associated with nutritional value [34], providing meals in a form as close as possible to a normal diet is important for maintaining the enjoyment of eating (e.g., through appetizing food appearance and texture). Previous studies have demonstrated that in addition to swallowing ability, oral health affects decisions regarding food forms in hospitalized patients capable of oral feeding [15, 35]. In patients under palliative care, the shorter the life expectancy, the more difficult oral self-care appears to become and the higher the frequency of dry mouth, gingival bleeding, and dysphagia [36]. On the contrary, the present study results suggested that swallowing ability and oral health are associated with oral intake and food form decisions, independent of time to death. Therefore, providing palliative care to terminal cancer patients, who tend to have deteriorated oral health [16, 17], with appropriate dental interventions to maintain or improve their oral health could help maintain their enjoyment of eating.

This study has limitations. First, in this cross-sectional study, the participants were limited to terminal cancer patients who died after receiving palliative care. Some patients receiving palliative care may be discharged from the hospital. Therefore, the results of this study may not be applicable to all patients with terminal cancer eligible for palliative care. Second, in this study, we aimed to comprehensively evaluate terminal cancer patients who died after receiving palliative care; therefore, we did not evaluate the cancer types. Previous studies have shown that patients with head and neck cancer or gastric cancer have increased difficulty with oral intake [37]. Considering these reports, evaluating the cancer type in this study might have helped clarify the systemic and oral factors that influence the decision of oral intake in terminal cancer patients. Third, appetite was not evaluated in this study. Poor appetite is common in terminal cancer patients approaching death [30, 31]. From the perspective of QOL, forcing oral feeding on patients who do not express a desire to eat is counterproductive. If appetite had been included in the factors examined in this study, we may have discovered oral factors that affect appetite, which could have further clarified the role of dentistry in maintaining QOL as part of a multidisciplinary palliative care approach for terminal cancer patients. Furthermore, this study was a single-center cross-sectional study. The results suggested that oral health impacts the nutritional method in terminal cancer patients. However, we did not determine the effect of the modified nutritional practices on the QOL and outcomes prior to the terminal disease stage. Moreover, which dental interventions were effective in improving the nutritional methods was still unclear. For terminal cancer patients, ambitious treatments requiring a significant time duration may be impractical. However, certain dental interventions should be provided. Based on the aforementioned factors, in future, longitudinal or interventional studies are needed to clarify the effect of modified nutritional practices with appropriate assessment for QOL and the type of dental interventions that are feasible and can effectively maintain oral feeding in terminal cancer patients.

## Conclusion

Based on their general condition and oral health, the terminal cancer patients receiving palliative care who participated in this study showed discrepancies in their provided and recommended nutritional methods. These discrepancies were caused by the lack of an appropriate comprehensive assessment of oral health status. Therefore, in the terminal cancer patients, oral assessment by dental professionals as part of a multidisciplinary approach may contribute to better selection of the appropriate nutritional intake methods. When considering the nutritional method for patients receiving palliative care, good swallowing ability and long prognosis are important to decide whether sufficient nutrition can be obtained by oral feeding alone. Furthermore, when oral feeding is possible, oral health, long-term prognosis, and swallowing ability need to be considered when deciding whether to use a higher-level food form.

## Data Availability

Not applicable.

## Declarations

### Disclaimer

The funders had no role in the design of the study; in the collection, analysis, or interpretation of data; writing of the manuscript; or in the decision to publish the results.
